# Multiple Organ Failure Due to Leukostasis Caused by Rapidly Progressive Hyperleukocytosis: A Case Report

**DOI:** 10.7759/cureus.88871

**Published:** 2025-07-27

**Authors:** Takanori Ohno, Tasuku Takahashi, Kazunori Murai, Hiroshi Nashiki

**Affiliations:** 1 Emergency Medicine, Iwate Prefectural Central Hospital, Morioka, JPN; 2 Nephrology and Rheumatology, Iwate Prefectural Central Hospital, Morioka, JPN; 3 Hematology, Iwate Prefectural Central Hospital, Morioka, JPN; 4 Emergency Medicine, National Health Insurance Hanamakishi Ishidoriya Medical Center, Morioka, JPN

**Keywords:** acute myeloid leukemia (aml), blast cells, hematologist collaboration, hydroxyurea, hyperleukocytosis (hl), leukapheresis, leukostasis, monocyte proliferation, myelodysplastic syndrome (mds), vascular occlusion

## Abstract

Myelodysplastic syndrome (MDS) is common in older adults and progresses to acute myeloid leukemia (AML) with a poor prognosis. Here, we present the case of a 71-year-old man undergoing treatment for MDS who was brought to the hospital for emergency care after suddenly becoming disoriented while on a trip. Laboratory results revealed a markedly increased white blood cell count (468,480/μL) with a predominance of blast cells (89%), and he was diagnosed with hyperleukocytosis (HL) and leukostasis associated with acute conversion to AML (probably M4). After surviving two cardiac arrests, the patient was deemed not strong enough to survive another cardiopulmonary arrest. A decision was made to withdraw from invasive treatment in the acute phase, and the patient died within eight hours of arrival at the hospital. It is challenging to determine whether the cause of vascular occlusion is a thrombus or a blast cell. Generally, when leukostasis is associated with HL and monocyte proliferation, occlusion by blast cells is the most common cause. Leukostasis is caused by the adhesion of intravascular blast cells and increased blood viscosity and is fatal, particularly in the respiratory and central nervous systems. Treatments include hydroxyurea/low-dose chemotherapy and leukapheresis; however, evidence of their ability to improve early mortality is lacking. In older patients, family opinion plays a vital role in treatment decisions, and collaboration with hematologists is essential.

## Introduction

Myelodysplastic syndrome (MDS) is common in both the United States and Japan, with a median age at diagnosis of 76 years, making it particularly prevalent among the older population [1,2]. The overall annual incidence rate in the United States is 3.4 per 100,000 individuals, with a higher rate in men (4.5 per 100,000) than in women (2.7 per 100,000). Among racial groups, the incidence is generally higher in Caucasians (3.5 per 100,000) than in Asians (2.6 per 100,000) [1]. A similar trend has been observed in Japan, with incidence rates of 3.8 per 100,000 in men and 2.4 per 100,000 in women [2].

MDS is a neoplastic disorder characterized by impaired hematopoiesis, morphological abnormalities of blood cells, and peripheral cytopenias, particularly thrombocytopenia, resulting from abnormal proliferation and apoptosis of immature hematopoietic cells. These features lead to significant complications such as anemia and hemorrhage associated with thrombocytopenia, as well as infections due to dysfunctional blood cells.

Approximately 30% of patients with MDS eventually progress to acute myeloid leukemia (AML), which is defined by the presence of more than 20% blast cells in the bone marrow [3]. Although the prognosis after progression to AML is generally poor, the leading causes of poor outcomes in most MDS patients are bleeding and infections, which are complications of the underlying disease itself [4,5]. In this report, we present a case of an MDS patient with acute transformation that led to death due to multiple organ failure.

## Case presentation

A 71-year-old man suddenly collapsed and required emergency medical assistance while waiting for the bus to return home after going sightseeing with his wife. He complained of loss of appetite the previous day. Upon contact with the emergency medical team, his blood pressure was 110/89 mmHg, his heart rate was 114/min, his consciousness was clouded, and both pupils were dilated to 5 mm. Based on these findings, particularly impaired consciousness and bilateral pupil dilation, the patient was suspected to have had a stroke and was transported to the hospital.

**Figure 1:**
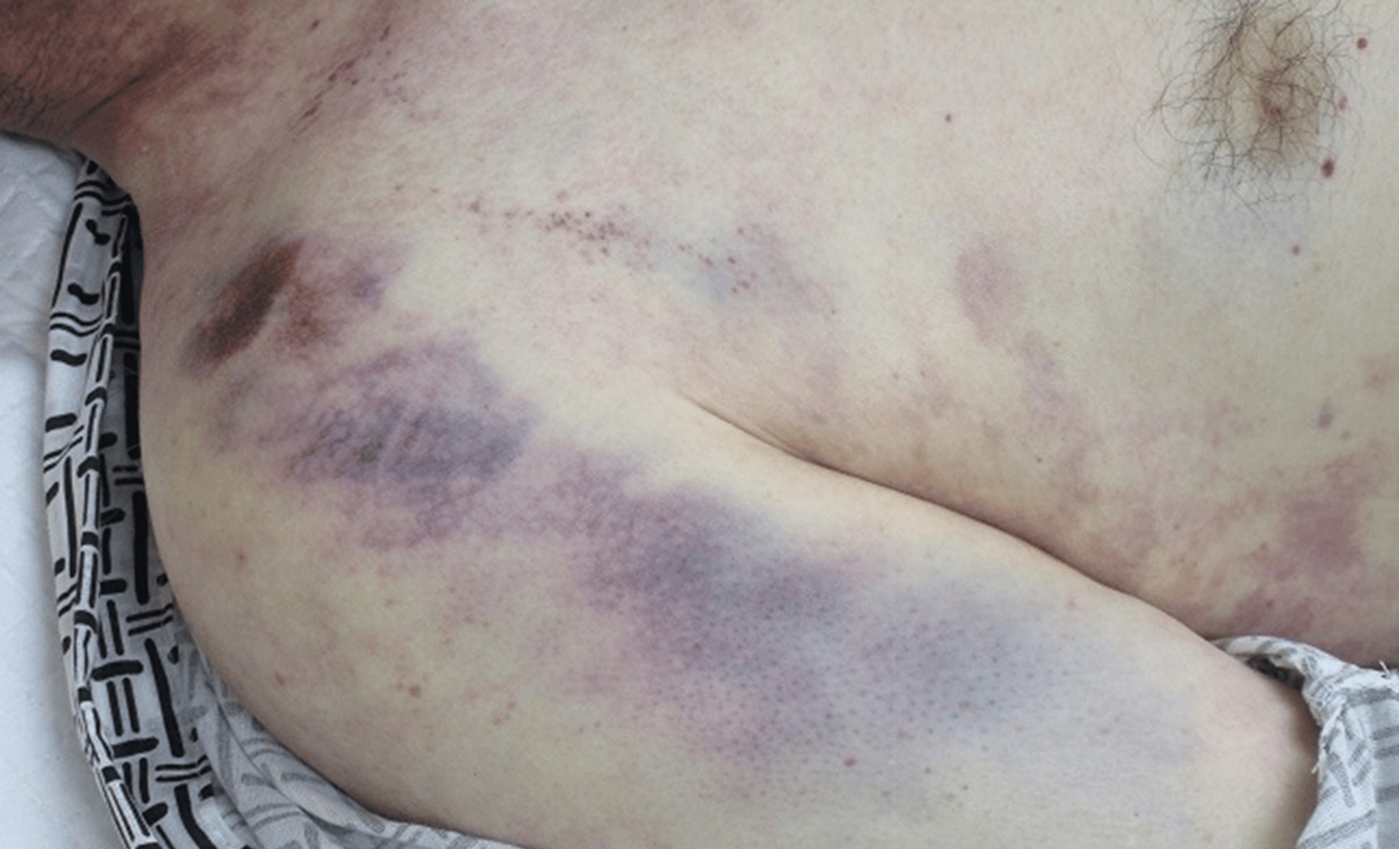
Subcutaneous bleeding around the right shoulder and in the lateral abdomen A large area of subcutaneous hemorrhage centered on the right shoulder, potentially caused by a fall. The formation of hemorrhages was considered associated with the DIC state. DIC: disseminated intravascular coagulation

Upon visiting the hospital, in addition to the above findings, a physical examination revealed abdominal breathing, a sunken root of the tongue, and a lack of a light reflex in the pupils. Extensive subcutaneous bleeding around the right shoulder and lateral abdomen was also observed, likely due to a fall (Figure 1).

The patient was initially judged to be in an unstable respiratory condition, and assisted ventilation was initiated. However, as the carotid pulse was no longer palpable after a few minutes, chest compressions were initiated, and 1 mg of intravenous adrenaline was administered. The heartbeat resumed after 2 minutes; thus, ventilatory management was initiated following tracheal intubation, and close examination was continued. Although the patient was transported with suspected stroke, head computed tomography revealed no abnormal findings. No findings other than splenomegaly were observed on the trunk.

The only information his wife could provide was that he was receiving treatment for MDS and that his primary physician had indicated the condition had stabilized. We also contacted the primary care physician's office; however, since he was unavailable, we were unable to obtain detailed information, such as where he received his medications or the results of his recent blood tests. Laboratory examination revealed the following results (Table 1).

**Table 1:** Laboratory data Arterial blood gas showed worsening oxygenation even with 10 L of oxygen; elevated urea nitrogen and creatinine and progressive metabolic acidosis indicated that renal failure was also progressing. The elevation of D-dimer and the decrease of Fib indicated rapid thrombus formation.

Test	Values	Reference range
Red blood cells (×104/μL)	2.46	4.35–5.55
Hemoglobin (g/dL)	7.8	13.7–16.8
Hematocrit (%)	30.1	40.7–50.1
Mean corpuscular volume (fl)	122.4	83.6–98.2
Platelet (×104/μL)	77	158–348
White blood cells (/μL)	468.48	3.30–8.60
Blast (%)	89	-
Promyelocyte (%)	0	-
Myelocyte (%)	0	-
Metamyelocyte	0	-
Monocyte (%)	5	2–12
Segment cell (%)	2	22–72
Stab cell (%)	0	0–18
Lymphocyte (%)	4	25–48
International normalized ratio	2.4	0.91–1.12
Activated partial thromboplastin time (sec)	55.1	24.0–39.0
Fibrinogen quantity (mg/dL)	117	200–400
D-dimer (μg/dL)	274.1	0.00–1.00
Total bilirubin (mg/dL)	0.62	0.40–1.50
Aspartate aminotransferase (U/L)	238	13–30
Alanine aminotransferase (U/L)	41	10–42
Lactate dehydrogenase (U/L)	6804	124–222
Alkaline phosphatase (U/L)	203	38–113
Creatine kinase (U/L)	2335	59–248
Urea nitrogen (mg/dL)	31.5	8.0–20.0
Creatinine (mg/dL)	2.73	0.65–1.07
C-reactive protein (mg/dL)	15.91	0.00–0.14
Procalcitonin (ng/dL)	2.3	0.00–0.05
Arterial blood gases (10L O2)		
pH	6.947	7.35–7.45
PaCO2 (mmHg)	30.5	35–48
PaO2 (mmHg)	56.6	83–108
HCO3 (mmol/L)	6.3	21–28
Base excess (mmol/L)	-24.1	-1.5-+3.0
Sodium (mmol/L)	137	136–146
Potassium (mmol/L)	5.4	3.4–4.5
Chloride (mmol/L)	107	98–106
Glucose (mmol/L)	152	70–105
Lactate acid (mmol/L)	135	0.5–1.5

Blood analysis indicated that 89.0% of the cells were blasts. Furthermore, arterial blood gas analysis showed worsening oxygenation despite administering 10 L of oxygen. Elevated urea nitrogen and creatinine levels and progressive metabolic acidosis indicated the progression of renal failure. The elevation of D-dimer levels and the decrease in Fib levels indicated rapid thrombus formation. These available test results indicated the occurrence of acute conversion to acute leukemia due to MDS, and concurrently, the patient was in a state of multiple organ failure due to the rapid progression of disseminated intravascular coagulation (DIC).

He then survived a second cardiopulmonary arrest. After explaining these observations and that the patient was not strong enough to survive another cardiopulmonary arrest, a decision was made to withdraw from invasive treatment in the acute phase. Ultimately, the patient died within 8 hours of arrival at the hospital.

**Figure 2:**
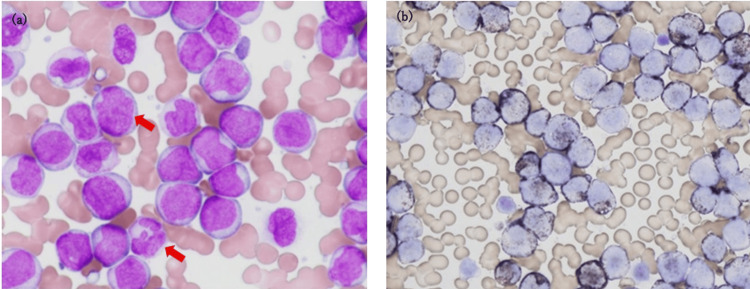
(a) Peripheral blood smear (×1000) using MG stain. (b) Peroxidase staining using the DAB method (a) Numerous mononuclear cells with high N/C ratios are also observed. These cells are morphologically consistent with the blasts. Some blasts exhibit irregular nuclear contours (red arrows). No gold rods are observed. (b) Blasts observed by MG staining are positive for DAB staining. Based on these findings, blasts were identified as myeloid cells. Non-specific esterase staining was not performed. MG: May-Grünwald-Giemsa, DAB: 3,3'-diaminobenzidine, N/C: nuclear-to-cytoplasmic

Additional test results later revealed that the blast cells were partially stained dark brown by 3,3′-diaminobenzidine (DAB) staining and exhibited positivity for granulocyte lineage markers (Figure 2a-2b), confirming a myeloid origin. Additionally, flow cytometric (FCM) analysis demonstrated positivity for CD13 (51.9%), CD33 (99.5%), and myeloperoxidase (53.9%) along with the expression of monocytic markers CD4 (72.7%) and CD14 (56.2%), suggesting a possible bone marrow monocytic lineage (M4). However, as nonspecific elastase staining was not performed to establish a definitive diagnosis, the French-American-British (FAB) classification of M4 was considered more likely.

## Discussion

In this report, the patient's pupils were dilated from the time of transport, and findings such as tongue deposition and abdominal breathing that became apparent after the patient arrived at the hospital were considered stroke-specific symptoms. Extensive subcutaneous hemorrhage was observed, and coagulopathy was suspected. Blood tests also revealed thrombocytopenia, and thrombotic damage to the respiratory organs, central nervous system, and kidneys was suspected due to hypercoagulability associated with DIC.

Notably, in a previous literature report of an autopsy case of acute conversion from MDS to AML that resulted in multiple organ failure, blast cells rather than thrombi occluded the blood vessels in various organs [6]. In the present case, the white blood cell count was also increased to 468,480, with 89.0% of blast cells visually detected.

This elevated leukocyte count is referred to as hyperleukocytosis (HL), a condition in which the leukocyte count exceeds 100,000/μL; it is associated with poor prognostic factors such as leukostasis, DIC, and tumor lysis syndrome.

HL occurs more frequently in acute leukemia than in chronic leukemia, being observed in 5-13% of adults with AML and in 10-30% with acute lymphocytic leukemia (ALL) [7]. Although ALL is more common than AML, AML is more frequently associated with leukostasis and requires urgent treatment. This is attributed to endothelial dysfunction caused by blast cell adhesion to the vascular endothelium as well as increased blood viscosity, as the average cell volume in AML is approximately twice that in ALL [8,9]. Obstruction can occur in any organ; however, when it involves the respiratory tract or central nervous system, the early prognosis is poor (20-40%) [10]. In this case, the family wanted to send the body home as soon as possible, so we could not propose an autopsy. However, based on the above, it is considered that, as in previous reports, vascular occlusion by the blast cells occurred, resulting in multiple organ failure.

AML often causes leukostasis subtypes M4 and M5 due to monocyte proliferation, as well as M3v, according to the FAB classification [11,12]. The FCM results of this case also indicated strong monocytic proliferation, and the DAB staining results suggested acute conversion to M4.

The prognosis for HL following this course is poor, with 8% survival at 24 hours after presentation and 25-50% survival at one week [8,9,13]. Therefore, patients with HL or leukostasis should receive multidisciplinary treatment in the intensive care unit. The treatment of DIC due to blood clots and vascular occlusion caused by blast cell stasis differs. The initial approach is to avoid blood transfusions and diuretics that may increase blood viscosity while administering fluid loading to alleviate stasis. Subsequently, treatment of the underlying malignancy includes hydroxyurea/low-dose chemotherapy, leukocyte therapy, radiation therapy, and corticosteroids targeting the causative hematologic malignancy [14]. However, hydroxyurea/low-dose chemotherapy and leukapheresis, commonly used in many centers, have not been demonstrated to improve early mortality [15,16].

The poor prognosis of HL and leukostasis appears to be partly attributable to the older age of patients at onset. A previous report indicated that 42% of older patients newly diagnosed with AML decline treatment [17]. Patients tend to avoid aggressive treatment only after consultation with family members to determine the most appropriate option [14]. Thus, it is tough to draw the line for aggressive treatment due in part to the rapid progression of HL, and it is necessary to select a treatment that takes into account the patient's background, as many patients are older. When HL is suspected, collaboration with a hematologist is crucial for selecting the appropriate treatment.

## Conclusions

Complications strongly influence the prognosis of MDS. In the emergency department, leukostasis due to HL with acute transformation rarely occurs but may result in multiple organ failure. In such cases, the initial response must differentiate between thrombi related to DIC and vascular occlusions caused by blast cells. Once multiple organ embolization occurs due to blast cell emboli, survival becomes extremely difficult. Even when therapeutic intervention is possible, collaboration with hematologists is essential, as treatment selection must be tailored to the patient's background.
